# Genome-Wide Re-Sequencing Data Reveals the Population Structure and Selection Signatures of Tunchang Pigs in China

**DOI:** 10.3390/ani13111835

**Published:** 2023-06-01

**Authors:** Feifan Wang, Zonglin Zha, Yingzhi He, Jie Li, Ziqi Zhong, Qian Xiao, Zhen Tan

**Affiliations:** School of Animal Science and Technology, Hainan University, Haikou 570228, China; wff1195394551@163.com (F.W.);

**Keywords:** Tunchang pigs, re-sequencing, genetic diversity, selection signal, candidate genes

## Abstract

**Simple Summary:**

The Tunchang pig has characteristics such as tolerance of coarse feed, strong disease resistance, and delicious meat. In 2014, it was listed as one of the main populations of pigs in Hainan and included in the National Livestock and Poultry Genetic Resources Protection List. Protecting and developing the genetic resources of Tunchang pigs can effectively ensure the sustainable development of animal husbandry in Hainan Province. Whole-genome re-sequencing data were used to analyze the genetic diversity, population structure, and selection signals of Tunchang pigs. Compared to commercial pigs, Tunchang pigs have higher genetic diversity and are closer to indigenous Chinese breeds in terms of genetics. Through comprehensive analysis of multiple selection signals, candidate genes for meat quality, disease resistance, growth, and reproduction were identified.

**Abstract:**

Tunchang pig is one population of Hainan pig in the Hainan Province of China, with the characteristics of delicious meat, strong adaptability, and high resistance to diseases. To explore the genetic diversity and population structure of Tunchang pigs and uncover their germplasm characteristics, 10 unrelated Tunchang pigs were re-sequenced using the Illumina NovaSeq 150 bp paired-end platform with an average depth of 10×. Sequencing data from 36 individuals of 7 other pig breeds (including 4 local Chinese pig breeds (5 Jinhua, 5 Meishan, 5 Rongchang, and 6 Wuzhishan), and 3 commonly used commercial pig breeds (5 Duorc, 5 Landrace, and 5 Large White)) were downloaded from the NCBI public database. After analysis of genetic diversity and population structure, it has been found that compared to commercial pigs, Tunchang pigs have higher genetic diversity and are genetically close to native Chinese breeds. Three methods, F_ST_, θπ, and XP-EHH, were used to detect selection signals for three breeds of pigs: Tunchang, Duroc, and Landrace. A total of 2117 significantly selected regions and 201 candidate genes were screened. Gene enrichment analysis showed that candidate genes were mainly associated with good adaptability, disease resistance, and lipid metabolism traits. Finally, further screening was conducted to identify potential candidate genes related to phenotypic traits, including meat quality (*SELENOV*, *CBR4*, *TNNT1*, *TNNT3*, *VPS13A*, *PLD3*, *SRFBP1*, and *SSPN*), immune regulation (*CD48*, *FBL*, *PTPRH*, *GNA14*, *LOX*, *SLAMF6*, *CALCOCO1*, *IRGC*, and *ZNF667*), growth and development (*SYT5*, *PRX*, *PPP1R12C*, and *SMG9*), reproduction (*LGALS13* and *EPG5*), vision (*SLC9A8* and *KCNV2*), energy metabolism (*ATP5G2*), cell migration (*EPS8L1*), and olfaction (*GRK3*). In summary, our research results provide a genomic overview of the genetic variation, genetic diversity, and population structure of the Tunchang pig population, which will be valuable for breeding and conservation of Tunchang pigs in the future.

## 1. Introduction

Pork has a broad consumer market and has become the world’s second most consumed meat product due to its high production, good taste, and easy processing [1,2]. According to statistics from the Food and Agriculture Organization of the United Nations (FAO), pork production accounted for more than 30% of total meat production in 2021, reaching a level of 120 million tons. China has a history of consuming pork for thousands of years and is currently the world’s largest producer and consumer of pork [3]. Chinese pork production capacity has reached 42.102 million tons, and pork consumption accounts for 73.9% of total meat intake [4,5]. However, the current domestic production and high cost still cannot meet the national demand for pork, so it is still necessary to import some pork from abroad to meet domestic market demand. At the same time, the domestic pork market in China is also undergoing changes. With the booming economy, more and more people are pursuing better-quality local pig breeds [6].

As one of the native pig population in China’s southernmost region, Tunchang (TC) pigs are well known for delicious meat, strong reproductive capacity, high stress resistance, and ability to tolerate coarse feed [7]. In February 2014, TC pig population was listed as a major group of local pigs in Hainan Province and was included in the National Livestock and Poultry Genetic Resources Protection list. As an independent group, TC pigs have a high value, not only as a branded breed for local livestock development to boost the local economy but also for its rich genetic diversity resources. To protect and develop the genetic resources of the TC pig, Hainan Province has approved several protection projects for TC pigs since 2015. The aim is to establish a TC pig core group for conservation and breeding, carry out genetic resource core group sorting and resource collection, and effectively promote the protection and development of TC pig resources.

Livestock exhibits rich diversity in appearance, reproductive ability, growth, adaptability, and other aspects to adapt to the environment and meet human needs. China has a significant advantage in the genetic resources of domestic pigs because it has over one-third of the world’s total pig breeds [8]. With its vast territory and diverse geographical environment, China has a wide variety of local pig breeds that have evolved genetic adaptations to different environmental conditions as people have migrated and domesticated them [9]. For example, the different temperatures and altitudes in the north and south have created differences in the size, hair, and physiological regulation of pig populations [10]. Chinese pig breeds also differ significantly from those in Western countries such as might be found in Europe. Apart from distinguishing them by appearance and size, population structure diagrams drawn using genomic data can also intuitively differentiate between the two [11].

Compared with another local pig breed in Hainan Province, the Wuzhishan (WZS) pig, there is little research on the TC pig. Therefore, to fully develop and utilize the breeding resources of TC pigs, it is necessary to accurately assess the genetic diversity and population structure of the existing TC pig population. Currently, the use of high-throughput sequencing technology to explore genetic markers at the individual or population level to investigate genetic diversity and reveal the genetic basis of certain animal species’ specific traits is already mature and widespread, as it has been applied in pigs, chickens, cows, sheep, and other animals [12,13,14,15]. Therefore, this study aims to explore the genetic diversity and population structure of TC pigs by utilizing whole genome re-sequencing data from 10 male individuals with no relationship within three generations of animal genealogy, collected from a conservation farm in the Hainan Province. Our results can serve as data and information for the current genetic resource statistics and evolutionary history of the TC pig and serve as a guide for conservation breeding efforts to effectively utilize this population as a valuable tool.

## 2. Materials and Methods

### 2.1. Sample Collection and DNA Extraction

10 male TC individuals’ whole blood samples were sampled from the farm of TC pig (Tunchang Tianzhihong Ecological farm Co., Ltd., Tunchang, China). Following the library construction protocol, the genomic DNA of each sample was extracted from blood tissue using a commercial kit (Tiangen Biotech Co., Ltd., Beijing, China).

### 2.2. Sequencing and Published Data Download

Qualified libraries were sequenced on the Illumina NovaSeq platform (Illumina, San Diego, CA, USA), generating paired-end reads of 150 bp in accordance with the manufacturer’s protocol. Whole-genome high-coverage sequencing was performed on these 10 individuals with an average coverage depth of 10×. In addition, to better reflect the diversity level of the TC pig population, re-sequencing data from 36 individuals of 7 other pig breeds were obtained from a public database (https://www.ncbi.nlm.nih.gov/sra, accessed on 18 April 2022) and combined with the TC pigs’ re-sequencing data for population analysis. Therefore, based on the geographic distribution described in Supplementary Figure S1, this study divided the population into Tunchang pigs (TC) and Wuzhishan pigs (WZS) in southern China; three other indigenous Chinese pig breeds, including the Rongchang pig (RC), in southwestern China; the Jinhua pig (JH) and Meishan pig (MS) in eastern China; and three commonly used commercial pig breeds, including the Duroc pig (DU), Landrace pig (LR), and Large White pig (LW). The summary of the downloaded sequencing data, sample sizes, and other information can be found in Supplementary Table S1.

### 2.3. Data Quality Control and Genotyping

The raw unprocessed data were merged and quality controlled (QC) using the software program FASTP (version 0.20.1, https://github.com/OpenGene/fastp), which ensured high-quality data by trimming adapters and removing low-quality nucleotides, unknown nucleotides (NS), and reads containing more than 10% N. Filtered reads from all individuals were aligned to the pig reference genome Sus scrofa11.1 using the Burrow–Wheeler aligner software program (BWA, version 0.7.17) [16]. The software program Sambamba (version 0.8.2, https://github.com/biod/sambamba) was used to discard duplicates and remove unmapped or low mapping quality score reads from the alignment results. The remaining reads were defined as good reads and used for further analysis. All parameters were set to default. Single-nucleotide polymorphism (SNP) calling was performed using GATK (V4.0.3.0) and filtered according to the parameters established by Xu et al. [17]. All sex chromosomes and “NW_“chromosomes were deleted from the VCF file; only 18 autosomes chromosomes were retained for subsequent analysis. SNP quality control was then performed using VCFTOOLS (v 0.1.16) with the parameters “–minDP 4 –max-missing 0.5 –minQ 30 –maf 0.05 –minGQ 10,” which meant that only sites with depth ≥ 4, missing rate ≤ 0.5, minor allele frequency ≥ 0.05, and calling quality ≥ 30 were retained for subsequent analysis. The missing genotypes were imputed using the Stitch package in R language [18], which only requires data with ultra-low coverage to perform imputation [19]. Finally, functional annotation was performed using the ANNOVAR software package (version 7 June 2020) [20,21], and SNPs were classified according to the pig gene annotation file downloaded from the NCBI database (GCF_000003025.6_Sscrofa11.1_genomic.gff.gz).

### 2.4. Analysis of Genome Diversity and Inbreeding

By completing the above steps, a total of 21,912,205 common SNP sites were obtained to infer the genetic information of these 8 pig populations. First, nucleotide diversity (θπ) [22], which can reflect nucleotide differences, was calculated using the software program VCFTOOLS (v0.1.16). Then the population differentiation index (F_ST_) was calculated using VCFTOOLS (v0.1.16), which reflects the degree of differentiation between the tested populations based on their genetic polymorphism data [23]. The linkage disequilibrium (LD) values for all populations were calculated using the software program PopLDdecay (v3.41) with default parameters, which were calculated based on the squared correlation (r2) between SNPs [24]. Data filtering was performed using parameters „-MAF 0.05 -MAX-Miss 0.5” before analysis. The average r2 value between pairwise SNPs was estimated under the default maximum distance, and the overall average value was obtained. Plink (v1.9) was then used to calculate runs of homozygosity (ROH) segments for each individual, and the mean ROH segment value for each population was divided into seven categories: [100, 200] kb, [200, 300] kb, [300, 400] kb, [400, 500] kb, [500, 600] kb, [600, 700] kb, and ≥700 kb. The number of ROH segments in each of the seven categories for each population was then calculated. The coefficient of inbreeding (FROH) based on ROH was calculated by dividing the sum of the lengths of all ROH segments on autosomes by the physical total length of the pig autosomes (2265.77 cM in this study) [25]. The drawing F_ST_ heat map and FROH bar chart were inspired by Yang et al. [26].

### 2.5. Population Structure Analysis

First, the software program MEGA (v 7.0) was used to construct a population neighbor-joining (NJ) tree, which reflects the developmental relationships between individuals at the whole-genome level based on the identity-by-state (IBS) distance matrix data [27]. IBS was calculated using Plink (v 1.9) [28], and principal component analysis (PCA) was performed using the software program GCTA v 1.91.7 [29], followed by visualization using the ggplot2 package in R language. To visually represent the potential ancestral proportions or levels of admixture between individuals, the software program Admixture (v 1.3) was used to calculate the population genetic structure, and the population structure plot was generated using the Pophelper package in R language [30].

### 2.6. Selection Signature Detection of the Tunchang Pigs

Three genome selection feature detection methods were used to detect genome selection markers in TC, DU, and LR pigs. The three methods were the population differentiation coefficient method (F_ST_), polymorphism level statistics method (θπ), and cross-population extended haplotype homozygosity (XP-EHH). F_ST_ and θπ (DU or LR/TC) for TC and DU or LR breeds were calculated using VCFTOOLS (v0.1.16). The genome window and step size were set to 100 kb and 10 kb, respectively. Then log2 transformation was performed on θπ (DU or LR/TC) [26]. XP-EHH detects almost fixed or ongoing selection features by comparing haplotypes of different populations [31]. Selscan (v1.3.0) was used to calculate the XP-EHH value, and then the detected XP-EHH values were normalized [32]. The top 5% extreme values of each method were taken as potential candidate regions. Gene functional identification of the selected regions was performed based on the pig gene annotation file downloaded from the NCBI database (GCF_000003025.6_Sscrofa11.1_genomic.gff.gz). To further understand the potential biological functions and significance of the genes in these candidate regions, KOBAS (version 3.0, http://kobas.cbi.pku.edu.cn/) was used to analyze the richness of gene ontology (GO) terms and Kyoto Encyclopedia of Genes and Genomes (KEGG) pathways containing identified variants [33]. Only GO terms and KEGG pathways with Corrected *p*-Value *p* < 0.05 were further interpreted in this study. Finally, to understand the overlap information between the selected candidate regions and published QTL traits, pig QTL files were downloaded from the QTLdb database for comparison [34].

## 3. Results

### 3.1. Single Nucleotide Polymorphism Discovery

To study the genetic variation characteristics of TC, whole-genome re-sequencing was performed on 10 individuals using the Illumina NovaSeq platform. The statistical results of the sequencing data are listed in Supplementary Table S1, and the detailed information of the other 36 individuals’ re-sequencing data downloaded from the SRA database is listed in Supplementary Table S1, including three commonly used commercial breeds (5 DU, 5 LR, and 5 LW) and four indigenous Chinese breeds (5 JH, 5 MS, 5 RC, and 6 WZS). In total, 262.21 GB of raw data were obtained from the high-coverage sequencing data of 10 individuals, with an individual Q30 score of 93.83. After quality control using the software program FASTP, 114.85 GB of clean data were retained for further re-sequencing analysis.

**Figure 1:**
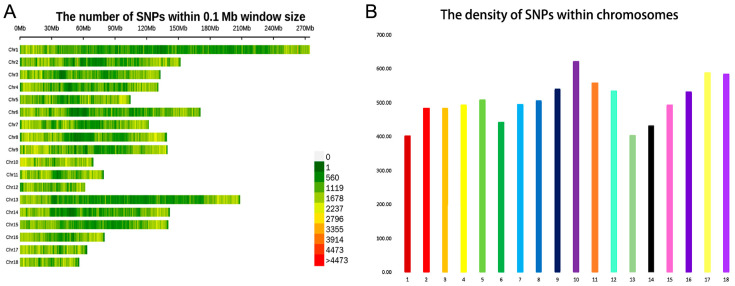
The distribution of SNPs detected in Tunchang pigs across chromosomes. (**A**) Genome-wide distribution of detected SNPs on 18 autosomes chromosomes for Tunchang pigs. The X-axis represents the number of SNVs (single-nucleotide variants). The Y-axis represents 18 autosomes chromosomes, calculated as the number of SNPs per 0.1 Mb. (**B**) Genome-wide density distribution of detected SNPs on 18 autosomes chromosomes for the Tungchang pigs. The X-axis represents 18 autosomes chromosomes. The Y-axis represents the number of SNPs per 0.1 Mb on a chromosome.

After strict quality control and filtering, a total of 12,595,393 SNPs were detected in TC. Then the annotated file of all detected SNPs in TC was obtained using the gene annotation file downloaded from the NCBI database. It was evident that the highest number of SNPs were found in the intergenic region (45.03%) and introns (42.37%). Only 0.553% of the SNPs were located in the exonic region, including 22,342 (32.09%) nonsynonymous SNPs and 44,152 (63.41%) synonymous SNPs (Supplementary Table S2). We also analyzed the number and density distribution of SNPs detected on each chromosome in TC. The results showed that the number of SNPs was highest on chromosome 1, reaching 1,290,415 (Figure 1A), while the SNP density was highest on chromosome 10 (Figure 1B). Overall, the distribution of these SNPs indicates that these variants are uniformly distributed on each chromosome. These potential functional SNPs provide important genetic resources for exploring the genetic structure and selection traits of TC.

### 3.2. Genetic Diversity

**Figure 2:**
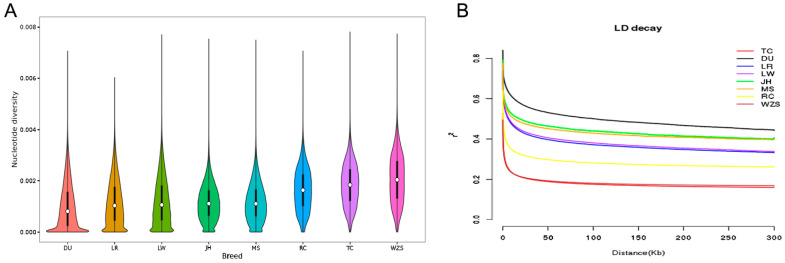
Genome nucleotide diversity and linkage disequilibrium decay of tested pig populations were analyzed in this study. (**A**) Genomic nucleotide diversity of all tested pig populations analyzed in this study. (**B**) Linkage disequilibrium decay of all tested pig populations analyzed in this study, denoted with one line for each population.

Population genetic diversity can be reflected by the θπ value, with lower diversity indicating a higher degree of selection in the population. As can be seen from Figure 2A, the three commercial pig breeds DU (0.00101), LR (0.00115), and LW (0.00186) have the lowest θπ values, while JH (0.00114) has the lowest value among the indigenous Chinese pig breeds, followed by MS (0.00116). TC (0.00184) and WZS (0.00205) have the highest θπ values.

LD analysis using r2 values to measure LD decay provides information about the overall diversity level of each test population. DU showed the highest level of LD among all tested populations, while TC and WZS had the lowest LD levels. In addition, LD decay in JH and MS populations from other regions of China was found to be above that of the commercial pig breeds LR and LW (Figure 2B). The results presented in the LD decay plot are consistent with the aforementioned θπ analysis, further indicating that the DU, JH, and MS populations may have undergone strong selection pressure (Figure 2B). The selection pressure on the RC, TC, and WZS populations was relatively small, consistent with the nucleotide diversity results.

**Figure 3:**
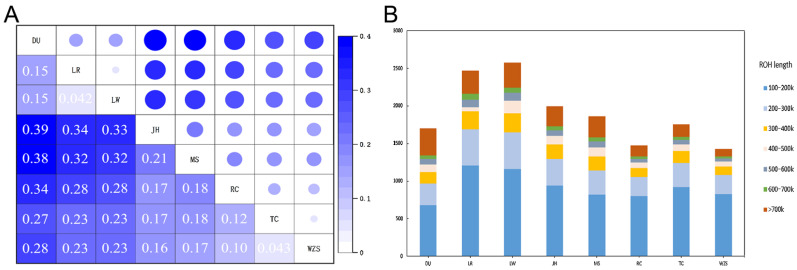
Genetic differentiation in each population. (**A**) Heatmap of F_ST_-weighted values are used to denote the genetic differentiation. (**B**) Columnar distribution of seven categories of ROH fragments of each breed.

To further investigate the degree of population differentiation between TC and other pig breeds, we estimated the F_ST_ values for all populations (Figure 3A). The genetic differentiation between TC and its three commercial pig breeds (0.23–0.27) was much higher than that between TC and other local Chinese pig breeds (0.043–0.18), with the lowest genetic differentiation observed between TC and WZS (0.043).

We calculated the inbreeding coefficient FROH based on ROH. Overall, the FROH of commercial pig breeds (0.460–0.549) was higher than that of local Chinese pig breeds (0.190–0.383). TC had the highest FROH (0.3827) among local Chinese pig breeds. These results suggest that existing breeding programs have to some extent avoided inbreeding in the TC pig population. Then the ROH segments were divided into seven categories (Figure 3B). We found that the number of ROHs in TC was moderate, ranking fifth, with ROHs in the [100, 200] kb range accounting for more than 52% of the total ROHs in TC. This may indicate that the probability of inbreeding in recent generations is extremely low.

### 3.3. Phylogenetic Relationships and Population Structure Analysis

**Figure 4:**
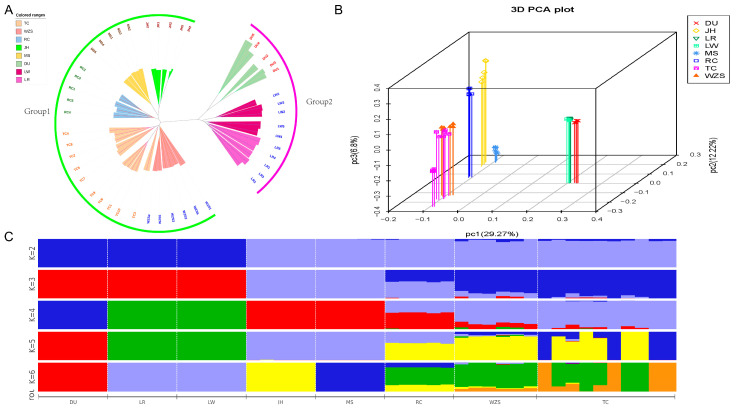
Population structure analyses of pig populations were conducted in this study. (**A**) Neighbor-joining tree constructed from SNV data among 8 breeds. (**B**) PCA plot the first three principal components for all breeds. (**C**) Admixture analysis at K= 2–6. (K = 3 is the best assumed genetic group).

To explore the genomic similarities and differences between TC and other tested populations, neighbor-joining (NJ) tree, PCA, and population structure analysis were conducted (Figure 4). In the NJ tree constructed using all individual data, it can be observed that all individuals are clustered under their respective breeds, indicating breed representativeness at the genetic level (Figure 4A). The clustering groups can be roughly divided into two groups: local Chinese populations (TC, WZS, JH, MS, and RC) and three commonly used commercial breeds (DU, LR, and LW), indicating significant genetic differences between commercial and local breeds. When clusters are located on different branches but originate from the same base, their relationship is close. Therefore, it can be seen that the TC population is close to the WZS population in terms of branching, indicating a close relationship between them.

PCA results are shown in Figure 4B. The first three principal components contributed to 29.27%, 12.22%, and 6.80% of the genetic variance, respectively. The results clearly separated the local Chinese populations from the commercial populations, with the commercial pig populations clustering more closely together, while the local Chinese breeds were also separated from each other by a certain distance. This may indicate that local Chinese breeds such as TC, WZS, JH, MS, and RC still maintain a relatively high level of population purity and are genetically distant from the commonly used commercial breeds. There was also clustering observed between DU, LR, and LW. From the figure, it can be seen that TC and WZS pigs are closely related, which is consistent with the NJ tree.

The results of the admixture analysis are shown in Figure 4C. Assuming K = 2 ancestral populations, eight test populations were divided into two populations, with one population consisting of the three commercial pig breeds and the other consisting of other Chinese indigenous pig populations. The cross-validation error test results showed that as K increased, pig breeds were gradually separated into different populations, with DU becoming an independent population when K = 4, LR and LW remaining in the same population, and there being obvious genetic admixture in Chinese indigenous pig populations, but they can be clearly divided into southern China (TC and WZS), eastern China (JH and MS), and southwestern China (RC). When K = 5, subgroups emerge within the TC population, with some TC individuals showing close genetic relationships with the WZS population. This finding is consistent with the PCA and NJ tree results mentioned above. The figure may indicate that there is frequent gene flow between pig populations, especially when K = 3–7. RC exhibits a mixture of JH, MS, TC, and WZS (Supplementary Figure S2). The value of K ranged from 2 to 12 (Figure 4C, Supplementary Figure S2). The cross-validation error test results showed that K = 3 represented the optimal number of ancestral populations (Supplementary Figure S2). In this case, TC and Western commercial pig breeds were distinguished, with only a certain proportion of Western and indigenous Chinese pig ancestry observed in the TC population. The TC population, which has been subject to multiple generations of selection, has developed a unique genetic structure and can be utilized as a unique genetic resource on Hainan Province in China.

### 3.4. Selection Signature Detection and Gene Annotation and Functional Analysis

**Figure 5:**
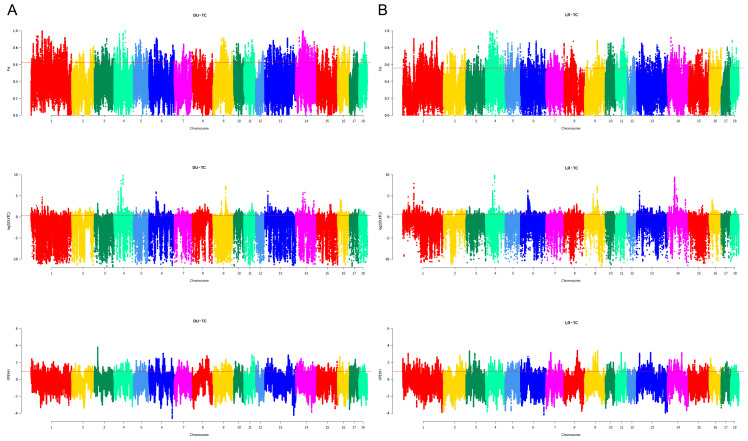
Genome-wide putatively selective signatures by F_ST_, θπ, and XP-EHH on 18 autosome chromosomes in the TC, DU, and LR populations. (**A**) Manhattan plot of weighted values of three methods between TC and DU. (**B**) Manhattan plot of weighted values of three methods between TC and LR. The X-axis represents 18 autosome chromosomes, and the Y-axis represents the weighted values of each method. Above the red line is the selected top 5% level.

**Figure 6:**
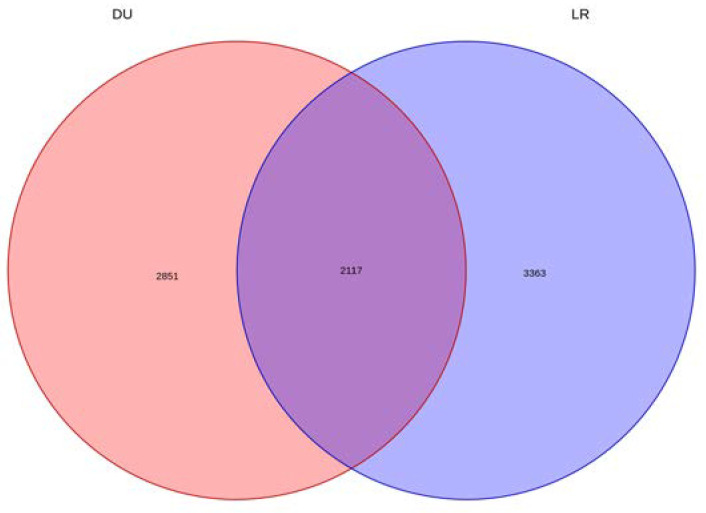
The Venn diagram shows the intersection of candidate regions by different populations. The left and right, respectively, represent the number of candidate regions detected between Tunchang and Duroc pigs, as well as between Tunchang and Landrace pigs.

As one of the unique pig populations on Hainan Province in China, the TC pig possesses its own excellent traits. To explore the genomic information of TC, the genomic features of TC were further compared with those of two typical commercial pig breeds (DU and LR). Three complementary methods (i.e., fixation index (F_ST_); nucleotide polymorphism level (θπ); and cross population extended haplotype homozygosity (XP-EHH)) were used to study the whole-genome selection signal. In order to reduce false positive candidate regions, regions with a top 5% threshold in at least two methods were selected as the selected region. Figure 5 shows the genomic distribution of candidate regions detected by different methods in TC and two typical commercial pig breeds. There were 4968 selection regions (threshold, 5%; F_ST_, 0.622899; θπ ratio, 0.469371498; XP-EHH: 0.934944; Supplementary Tables S4–S6, Supplementary Figure S3A) between the TC and DU populations, while there were 5480 selection regions (threshold, 5%; F_ST_, 0.560883; θπ ratio, 0.469371498; XP-EHH: 0.9196323; Supplementary Tables S7–S9, Supplementary Figure S3B) between TC and LR pigs. We found 2117 overlapping regions between the two sets of selected regions (Figure 6, Supplementary Table S10).

We annotated the obtained 2117 candidate regions and obtained 201 genes (Supplementary Table S11). Due to the inadequate functional annotation of the pig genome and the high genomic similarity and rich functional annotation of the human and pig genomes, candidate genes were transformed into human homologous genes through the BioMart of the Ensembl database. Gene enrichment analysis revealed significant enrichment (corrected *p* < 0.05) in 77 GO terms and 8 KEGG pathways (Supplementary Table S12). Significant GO terms included important pathways such as fatty acid metabolism, muscle calcium-binding protein complex, fatty acid biosynthesis, and diet-induced thermogenesis (Table 1). The significant KEGG pathways were mainly related to diseases (5 out of 8 pathways) and metabolic pathways (2 out of 8 pathways), including metabolic pathways, papillomavirus infection, protein glycosylation in cancer, taurine and hypotaurine metabolism, etc. (Table 2).

To investigate the overlap between previously published QTLs and candidate regions, the 36,725 QTLs divided into 698 different traits were downloaded from the Pig QTLdb database (version 49, 28 December 2022). We found that 2591 QTL data overlapped with the 2217 candidate regions (Supplementary Table S13). Notably, 1829 (70.6%) QTLs were associated with meat and carcass traits, indicating strong selection for meat quality in the breeding process of the TC breed.

To identify candidate genes more accurately, we used the 206 candidate regions that intersected between TC/DU and TC/LR based on F_ST_, θπ, and XP-EHH methods (Supplementary Table S14). In these regions, a total of 28 candidate genes were identified, including genes related to TC germplasm traits such as meat quality (SELENOV, CBR4, TNNT1, TNNT3, VPS13A, PLD3, SRFBP1, and SSPN), immune regulation (CD48, FBL, PTPRH, GNA14, LOX, SLAMF6, CALCOCO1, IRGC, and ZNF667), growth and development (SYT5, PRX, PPP1R12C, and SMG9), reproduction (LGALS13 and EPG5), and vision (SLC9A8 and KCNV2). These genes are distributed on chromosomes 1, 2, 4, 5, 6, 14, and 17 (Supplementary Table S15).

## 4. Discussion

To reveal the genetic variation information across the whole genome, there are currently techniques such as SNP array and whole-genome re-sequencing. Compared to SNP array technology, whole-genome re-sequencing has advantages such as richer genetic markers, more loci, and more representativeness. In this study, the Q30 (%) of the collected individuals were all greater than 93%, meaning that the percentage of bases with a quality value greater than or equal to 30 accounted for more than 93% of the total bases (the error rate of base calling is 1/1000). After calling high-quality reads for SNPs and filtering, a total of 12 595 393 SNPs were identified in the TC population, and it was found that the distribution density of SNPs on chromosome 10 was the most concentrated, indicating that chromosome 10 could be a target chromosome for further study.

The present study analyzed the whole-genome sequencing data of 10 TC pigs, 4 Chinese indigenous pig breeds (5 JH, 5 MS, 5 RC, and 6 WZS), and 3 commercial pig breeds (5 DU, 5 LR, and 5 LW). Generally, species with strong selection pressure have lower genetic diversity, while local breeds or varieties have higher levels of genetic diversity due to traditional breeding methods involving natural or random mating [35,36]. The results of θπ and LD indicate that TC pigs have a higher level of nucleotide polymorphism and lower selection pressure, consistent with the above research results, indicating the high level of genetic diversity in TC pig population. The F_ST_ between TC and other populations except for WZS ranges from 0.12 to 0.27, indicating a higher genetic similarity between TC pigs and local Chinese pig breeds. However, TC pigs still have some contamination by commercial pig bloodlines (Supplementary Figure S2).

A study reported that FROH can estimate inbreeding coefficients more accurately [37], so FROH was calculated for all tested populations. The results showed that FROH levels of different populations ranged from 0.190 to 0.549, and the FROH of commercial pigs was generally higher than that of local Chinese pig breeds. The FROH of TC pigs was the highest among local Chinese pig breeds, reaching 0.383, but the difference between TC and JH (0.380) and MS (0.381) was not significant. Jinhua pigs and Meishan pigs are famous Chinese pig breeds that have long-established national breeding centers for breed resource protection [38,39]. This indicates that the establishment of the TC breeding center has protected the breed resources to some extent and prevented inbreeding. The length of ROH segments can reflect the generation interval of common ancestors, and the shorter the segment, the more distant the interval [37]. The results showed that the ROH short segments (100–200 kb) of TC pigs accounted for more than 52% of the total segments, suggesting that there may have been more inbreeding in the early stage of breed formation, while the frequency of inbreeding in recent generations was relatively low [40].

The phylogenetic tree and principal component analysis results indicate that the population of TC pigs can be clearly distinguished from other pig breeds. They exhibit unique clustering and branching, indicating that the TC pigs’ genome has unique genetic characteristics after long-term domestication and breeding. Specifically, the population clustering in the phylogenetic tree and principal component analysis is highly consistent and has a certain correlation with the geographical distribution of the tested populations. All populations can be divided into local Chinese pig breeds and common commercial pig breeds, with local Chinese pig breeds further divided into eastern China (JH, MS), southern China (TC, WZS), and southwestern China (RC) based on their geographic distribution. The results of the admixture analysis are similar to those of the phylogenetic tree and principal component analysis. When the number of common ancestors is set to 2 (K = 2), it is easy to distinguish between commercial pigs and local Chinese pig breeds. This differentiation is maintained even when increasing the number of common ancestors. In this study, we found significant differences between southern Chinese pigs and eastern Chinese pigs, while southwestern Chinese pigs exhibit a mixed state between southern China and eastern China. These findings are consistent with the results reported by Huang et al. [41], who studied the population structure of indigenous Chinese pigs using customized BeadChips. This phenomenon of genomic differences based on geographic distribution suggests that geographic distance isolation may play an important role in population differentiation [42]. Since ancient times, gene flow between neighboring breeds has been inevitable. Therefore, the genetic similarity between TC pigs and WZS pigs at the genetic level may be due to their geographical proximity and artificial selection. However, the phenomenon of lineage separation after K = 5 indicates a certain level of differentiation within the TC population, suggesting the need for conservation breeding to purify and stabilize genetic information.

As one of the native pig population in the southernmost part of China, TC pigs have many excellent traits. Therefore, TC pigs must have unique selection labels on their genome. By comparing different breeds, candidate regions with high F_ST_ values, high θπ ratios, and high XPEHH values (top 5%) were selected. A total of 2117 overlapping regions were detected among the candidate regions, and 201 candidate genes were further identified in these regions. Gene ontology analysis showed that these candidate genes may play important roles in meat quality, immune regulation, and growth and development processes.

We detected 28 genes by comparing the overlapping genomic regions found with the F_ST_, θπ, and XP-EHH three methods, among which were genes related to the genetic characteristics of TC pigs, such as *SELENOV*, *TNNT1*, *TNNT3*, *VPS13A*, *PLD3*, and *SRFBP1*. The *SELENOV* gene is a member of the selenoprotein family and plays an important role in selenium metabolism [43]. Knockout of the *SELENOV* gene in mice increases fat accumulation, demonstrating that *SELENOV* can inhibit fat accumulation and promote energy consumption [44]. *TNNT1* and *TNNT3*, as muscle troponin *T* (*TnT*), regulate slow skeletal muscle *TnT* and fast skeletal muscle *TnT*, respectively. Proteomic studies have found that the expression of *TNNT1* and *TNNT3* proteins in Angus crossbred cattle and yaks is closely related to meat tenderness [45,46,47]. *VPS13A*, one of the lipid transfer proteins, plays an important role in lipid metabolism and membrane homeostasis [48]. The TC pig inhabits a tropical region, and heat stress can increase platelet count and blood viscosity, which increases the risk of cardiovascular and cerebrovascular thrombosis. However, the *VPS13A* gene can regulate platelet secretion and aggregation under heat stress conditions [49]. Triglycerides and phospholipids, as lipid oxidation precursors, can produce strong aroma compounds, which are key factors affecting the flavor of livestock and poultry meat [50,51]. Studies on Hu sheep and Dorper sheep have found that the important difference in flavor precursors between the two lies in lysophospholipids, and *PLD3* has been identified as one of the key genes regulating lysophospholipid metabolism [52]. The *SRFBP1* gene expresses a protein product called *p49/STRAP*, which is an auxiliary factor of serum response factor (*SRF*) that can assist *SRF* in regulating target genes [53]. Data indicate that *SRFBP1* can regulate early gene expression and cell metabolism, affecting cell skeleton morphology [54,55]. Studies using Mexican and American peoples and Yorkshire pigs as experimental materials have found that *SRFBP1* is a significantly correlated gene with fat deposition and is enriched in the insulin secretion pathway [56,57].

Several genes related to immune regulation were also detected, including *CD48*, *FBL*, *SLAMF6*, and *ZNF667*. *CD48* can promote the adhesion and activation of immune cells. For example, the interaction between *CD48* and *cis-CD2* can promote T-cell activation, while the interaction between *CD48* and *trans-CD2* can promote adhesion and co-stimulation of immunological synapses [58]. Research has shown that the expression of *CD48* plays an important role in hematopoietic recovery after sub-lethal irradiation in high-sunlight regions [59]. *FBL* and its mediated RNA 2′-O-methylation (Nm) modification can inhibit innate immunity and promote viral infection. Down-regulation of *FBL* can block viral infection of macrophages and inhibit viral infection. Knocking out *FBL* can also increase the expression of ISG and reduce viral invasion [60]. *SLAMF6* is one of the signaling lymphocytic activation molecules, which can affect T cell function in regulating cancer immunity, but the role of *SLAMF6* in T cell signaling is not clear yet and there are conflicting reports on whether *SLAMF6* enhances or inhibits T cell activity [61,62,63]. *ZNF667* (zinc finger protein 667, also referred to as Mipu1) participates in cell growth, apoptosis, oxidative stress, and lipid accumulation. In a study on laryngeal squamous cell carcinoma (*LSCC*), it was found that *ZNF667* is a tumor-suppressing gene [64,65,66,67,68]. *ZNF667* can regulate P-selectin expression in specific cells and leukocyte-endothelial adhesion after hypoxia [69].

The *SMG9* gene is essential for growth and development, and cells lacking *SMG9* exhibit global transcriptional dysregulation [70]. Deletion or mutation of the *SMG9* gene can result in severe developmental anomalies, including malformations or overgrowth of the brain, heart, face, or eyes, and developmental delay [71]. *LGALS13* is a placenta-specific gene that is crucial for the development and function of the human placenta [72]. *LGALS13* is highly expressed in the syncytiotrophoblast layer of the placenta and can induce apoptosis of cytotoxic T cells in maternal–fetal immunity [73]. The expression level of *LGALS13* is also associated with gender, with higher expression in early male placentas [74]. A GWAS study identified significant SNP loci within or near the *EPG5* gene related to complex birth traits, and the protein product of the *EPG5* gene plays a critical role in the autophagy pathway, which is beneficial for the differentiation of early embryonic stem cells [75,76]. *EPG5* is involved in regulating nucleic acid transport and connects the autophagy pathway with innate and adaptive immunity [77]. Therefore, these data may provide evidence for the role of candidate genes in immune response, growth and development, and reproduction in TC pigs.

## 5. Conclusions

This study analyzed the genetic diversity and population structure of TC pigs in comparison to commercial pig breeds and indigenous Chinese pig breeds. Based on genetic diversity parameters such as θπ, LD, F_ST_, and ROH, the TC pigs were found to have a high level of genome-wide genetic diversity. The population genetic structure analysis revealed that the TC pigs are closely related to indigenous Chinese pig breeds, particularly the WZS pig, which also originates on Hainan Island in China. The intersection of selection signals between the TC pigs and two commercial pig breeds identified potential genetic characteristics related to meat quality, disease resistance, growth, and reproduction. In summary, these findings contribute to a better understanding of the genetic information of the TC pig and provide data-based support for its conservation and improvement.

## Figures and Tables

**Table 1:** Top 10 GO terms enrichment analysis. The corrected *p* was calculated using Benjamini and Hochberg’s (1995) method.

Term	Input	Background	*p*	Coreected *p*^2^	Genes
Protein binding	129	11,779	2.37 × 10^−23^	3.70 × 10^−20^	*TMEM229B*, *BUB1B*, *PLK1*, etc.
Cytosol	58	5095	2.66 × 10^−09^	1.56 × 10^−06^	*ATP6V1D*, *RPS10*, *RPS16*, etc.
ATP binding	28	1463	2.99 × 10^−09^	1.56 × 10^−06^	*EARS2*, *BUB1B*, *PLK1*, etc.
Nucleoplasm	45	3630	2.39 × 10^−08^	9.32 × 10^−06^	*RPS10*, *UTP3*, *RPS16*, etc.
Plasma membrane	52	4619	3.43 × 10^−08^	1.07 × 10^−05^	*ATP6V1D*, *CDH12*, *RAB44*, etc.
Nucleus	55	5208	1.04 × 10^−07^	2.67 × 10^−05^	*CALCOCO1*, *UTP3*, *AHCTF1*, etc.
Extracellular exosome	31	2085	1.20 × 10^−07^	2.67 × 10^−05^	*ATP6V1D*, *RPS16*, *AHCTF1*, etc.
RNA binding	22	1366	2.78 × 10^−06^	5.43 × 10^−04^	*RPS10*, *UTP3*, *RPS16*, etc.
Cytoplasm	47	4624	3.38 × 10^−06^	5.86 × 10^−04^	*BUB1B*, *PLK1*, *MYO1E*, etc.
Mitotic cell cycle	7	134	8.53 × 10^−06^	1.33 × 10^−03^	*NEK4*, *BUB1B*, *PLK1*, etc.

**Table 2:** The significant KEGG pathways enrichment analysis. The corrected *p* was calculated using Benjamini and Hochberg’s (1995) method.

Term	Input	Background	*p*	Coreected *p*^2^	Genes
Metabolic pathways	21	1433	1.92 × 10^−05^	2.39 × 10^−03^	*NDUFAB1*, *ATP6V1D*, *EXTL3*, etc.
Cell cycle	6	124	5.73 × 10^−05^	4.48 × 10^−03^	*CCNB2*, *BUB1B*, *PLK1*, etc.
Thermogenesis	7	231	2.34 × 10^−04^	1.35 × 10^−02^	*NDUFAB1*, *LIPE*, *BMP8A*, etc.
Human papillomavirus infection	8	330	3.67 × 10^−04^	2.05 × 10^−02^	*ATP6V1D*, *HEYL*, *MAGI1*, etc.
Huntington’s disease	6	193	5.75 × 10^−04^	2.42 × 10^−02^	*NDUFAB1*, *DNAH3*, *ATP5A1*, etc.
Proteoglycans in cancer	6	203	7.43 × 10^−04^	2.52 × 10^−02^	*CD44*, *CDKN1A*, *ANK2*, etc.
Cushing’s syndrome	5	155	1.42 × 10^−03^	3.76 × 10^−02^	*AHR*, *WNT16*, *CDKN1A*, etc.
Taurine and hypotaurine metabolism	2	11	1.94 × 10^−03^	4.18 × 10^−02^	*GGT1*, *GGT5*

## Data Availability

The data were deposited in the National Center for Biotechnology Information’s Short Read Archive BioProject repository, under the accession number PRJNA953017.

## Supplementary Materials

The following supporting information can be downloaded at: https://www.mdpi.com/article/10.3390/ani13111835/s1, Supplementary Table S1: Overall Details of Individual Pigs. Supplementary Table S2: SNP exon annotation results in Tunchang pig population. Supplementary Table S3: Inbreeding coefficient of each breed. Supplementary Table S4: Potential Candidate Regions Detected by F_ST_ (TC VS. DU). Supplementary Table S5: Potential Candidate Regions Detected by Log2 (θπ) (TC VS. DU). Supplementary Table S6: Potential Candidate Regions Detected by XP-EHH (TC VS. DU). Supplementary Table S7: Potential Candidate Regions Detected by F_ST_ (TC VS. LR). Supplementary Table S8: Potential Candidate Regions Detected by Log2 (θπ) (TC VS. LR). Supplementary Table S9: Potential Candidate Regions Detected by XP-EHH (TC VS. LR). Supplementary Table S10: Candidate Regions Identified by More Than Two Methods (TC VS. DU) overlap (TC VS. LR). Supplementary Table S11: Gene Annotation of Candidate Regions in Table S10. Supplementary Table S12: The Results of Gene Ontology Enrichment Analyses and KEGG Enrichment Analyses. Supplementary Table S13: QTLs Overlapped with Candidate Selected Regions. Supplementary Table S14: Candidate Regions Identified by Three Methods (TC VS. DU) overlap (TC VS. LR). Supplementary Table S15: Gene Annotation of Candidate Regions in Table S14. Supplementary Figure S1: The origins of indigenous Chinese pig populations were tested in this study. Supplementary Figure S2: ADMIXTURE analysis was performed to estimate the optimum number of clusters (k) in the data set. (A) Cross validation errors for diverse k values. As shown, k = 3 minimizes the cross-validation error. (B) Ancestry of each sample using k = 2–12 clusters. Supplementary Figure S3: Venn diagram shows the overlap in the number of candidate regions detected by three methods. (A) Venn diagram shows the overlap in the number of candidate regions detected by three methods between Tunchang (TC) and Duroc (DU) pigs. (B) Venn diagram shows the overlap in the number of candidate regions detected by three methods between Tunchang (TC) and Landrace (LR) pigs.
