# Supplementary material for: Genome-Wide Re-Sequencing Data Reveals the Population Structure and Selection Signatures of Tunchang Pigs in China
Source: Animals (Basel). 2023 Jun 1;13(11):1835. doi: 10.3390/ani13111835 (PMC10252034; doi:10.3390/ani13111835)
Supplement: Supplementary file 1 [file animals-13-01835-s001.zip › animals-2365192-supplementary.pdf]

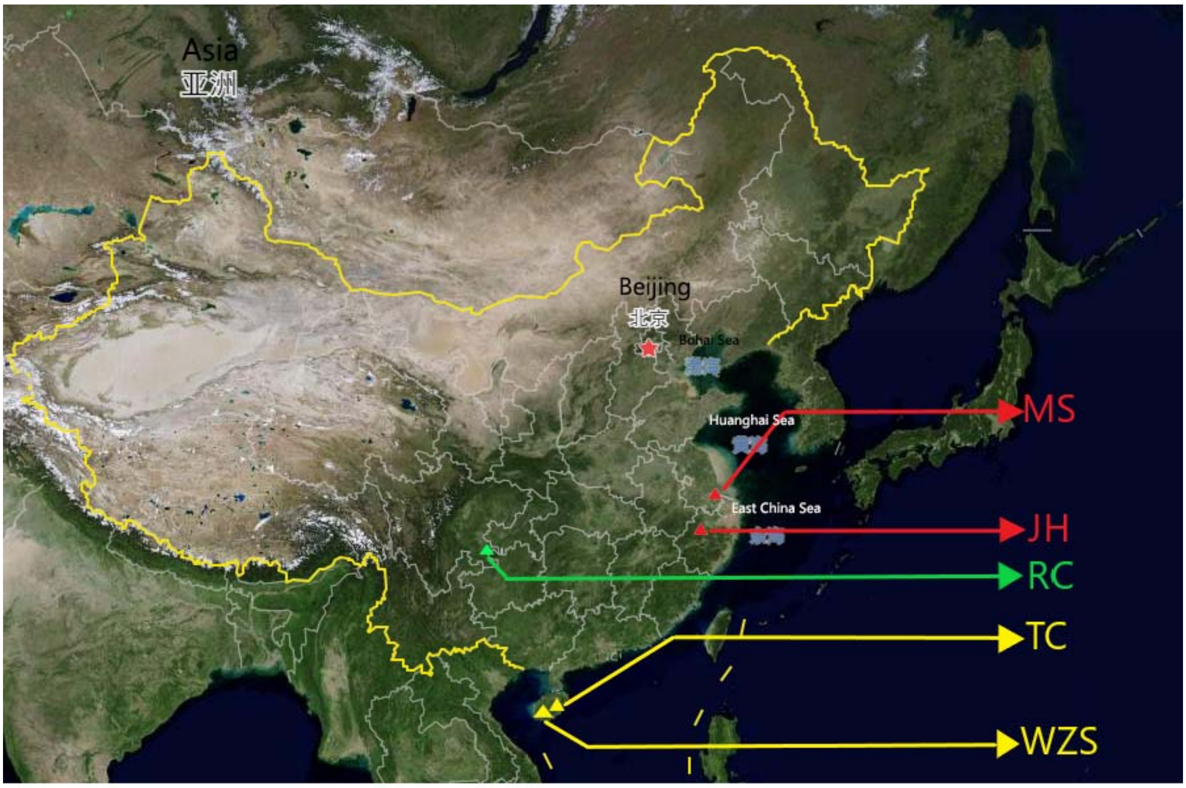

Figure S1. The origins of indigenous Chinese pig populations were tested in this study.

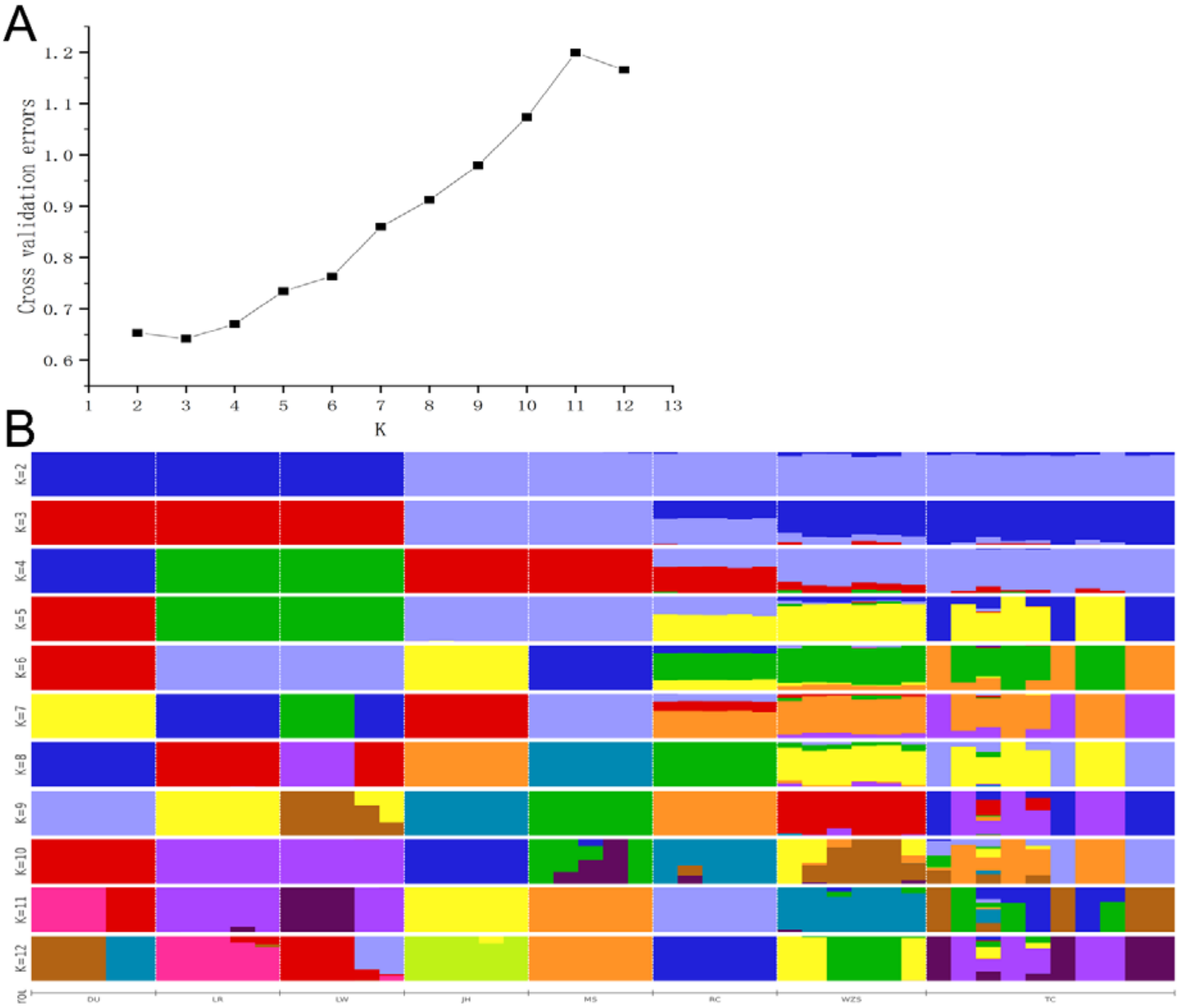

Figure S2. ADMIXTURE analysis was performed to estimate the optimum number of clusters (k) in the data set. (A) Cross validation errors for diverse k values. As shown, k = 3 minimizes the cross-validation error. (B) Ancestry of each sample using k = 2–12 clusters.

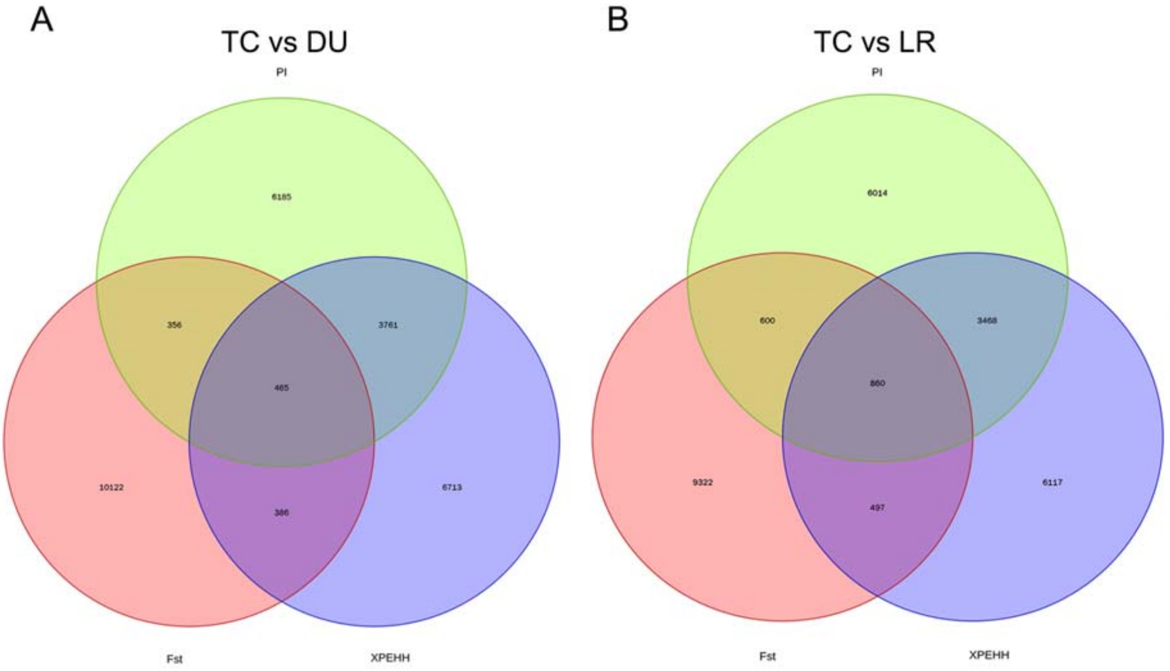

Figure S3. Venn diagram shows the overlap in the number of candidate regions detected by three methods. (A) Venn diagram shows the overlap in the number of candidate regions detected by three methods between Tunchang (TC) and Duroc (DU) pigs. (B) Venn diagram shows the overlap in the number of candidate regions detected by three methods between Tunchang (TC) and Landrace (LR) pigs.
